# Trials of Improved Practices (TIPs) to Enhance the Dietary and Iron-Folate Intake during Pregnancy- A Quasi Experimental Study among Rural Pregnant Women of Varanasi, India

**DOI:** 10.1371/journal.pone.0137735

**Published:** 2015-09-14

**Authors:** Siddharudha Shivalli, Ratan Kumar Srivastava, Gyan Prakash Singh

**Affiliations:** 1 Department of Community Medicine, Yenepoya Medical College, Yenepoya University, Mangalore, Karnataka, India; 2 Department of Community Medicine, Institute of Medical Sciences, Banaras Hindu University, Varanasi, India; 3 Division of Biostatistics, Department of Community Medicine, Institute of Medical Sciences, Banaras Hindu University, Varanasi, India; Centers for Disease Control and Prevention, UNITED STATES

## Abstract

**Background:**

Behavior Change Communications (BCC) play a decisive role in modifying socio-cultural norms affecting the perception and nutritional practices during pregnancy.

**Objective:**

To examine the effectiveness of ‘Trials of Improved Practices’ (TIPs) on dietary and iron-folate intake during pregnancy.

**Design:**

Community based quasi experimental study with a control group

**Setting:**

Four villages of *Chiraigaon* Community Development Block of Varanasi, India from May 2010 and recruited from August 2010. End line assessment, after 12 weeks of intervention, was completed in April 2011.

**Participants:**

Pregnant women in 13–28 weeks of gestation

**Intervention:**

TIPs was implemented in addition to ongoing essential obstetric care services in two villages through 3 home (assessment, negotiation and evaluation) visits and only assessment and evaluation visits in the other two control villages. Interpersonal communication, endorsing the active participation of family members and home based reminder materials were the TIPs based strategies. The effect of TIPs was assessed by comparing key outcome variables at baseline and after 12 weeks of intervention.

**Outcome Measures:**

Hemoglobin%, anemia prevalence, weight gain, compliance for iron-folate supplementation and dietary intake of calorie, protein, calcium and iron.

**Results:**

A total of 86 participants completed the study. At the end, mean hemoglobin levels were 11.5±1.24 g/dl and 10.37±1.38 g/dl in the TIPs and control groups, respectively. The prevalence of anemia reduced by half in TIPs group and increased by 2.4% in the control group. Weight gain (grams/week) was significantly (p<0.01) higher in TIPs group (326.9±91.8 vs. 244.6±97.4). More than 85% of the PW in TIPs group were compliant for Iron-folate and only 38% were compliant among controls. The mean intake of protein increased by 1.78gm in intervention group and decreased by 1.81 gm in controls (p<0.05). More than two thirds of PW in TIPs group were taking one extra meal and only one third of controls were doing the same.

**Conclusion:**

TIPs found to be an effective approach to improve the nutritional status of pregnant women in the study area. TIPs strategy could be further explored on larger sample representing different socio-cultural and geographical areas.

**Trial Registration:**

Clinical Trial Registry of India CTRI/2015/02/005517

## Background

Anemia continues to be a major public health problem in developing countries with its enduring ill effects on health, social and economic development. Although occurring at all stages of the life cycle, but pregnant women (PW) and young children are highly vulnerable. Affecting more than 500 million women in developing countries, it is a major cause of preventable morbidity and mortality among them [1]. Economic losses due to iron deficiency anemia alone are estimated at approximately $0.32 per capita or 0.6% of GDP [2].

Despite the high priority accorded to maternal and child health, anemia and poor gestational weight gain continue to prevail on the higher side in India. The impact of anemia control and nutritional supplementation programs has been dubious. Gestational anemia is a severe public health problem in India (prevalence > 40%, WHO 2001) and one of the leading causes of maternal death [3]. The reported prevalence of anemia among PW has increased from 49.7 percent in 1998–1999 to 58.7 percent in 2005–2006 (NFHS-3) [4]. Major reasons being increased demand, poor iron intake and improper dietary practices decreasing absorption of iron. Poor compliance of PW for Iron Folate (IFA) tablets has clearly been pointed out in nationwide surveys (NFHS-3 and DLHS-3) [4,5].

The scenario gets worse in the state of Uttar Pradesh state which accounts for the very high number of maternal deaths and Maternal Mortality Ratio in India. More than half of the PW in Uttar Pradesh are anemic, 26.3% have had at least 3 antenatal checkups and only 8.7% took 90 or more IFA tablets [4].

Existing evidence suggests that for every 1g/dl increase in hemoglobin among anemic PW, the risk of maternal death decreases by approximately 20%, making gestational Anemia control and prevention an important strategy to avert maternal deaths [6,7]. Therefore, better compliance for at least 100 IFA tablets and proper and adequate dietary intake are the issues in focus for the survival of PW.

At present in India, gestational anemia prevention and control is an integral part of Reproductive Maternal Neonatal Child and Adolescent Health (RMNCH+A) strategy under the ambit of National Health Mission [8]. It envisages imparting essential obstetric care to all the PW and encompasses at least 4 antenatal care (ANC) visits, including early registration and first ANC in first trimester along with physical and abdominal examinations, hemoglobin estimation and urine investigation, 2 doses of tetanus toxoid and consumption of IFA tablets (100 mg elemental iron and 0.5mg folate) for 100 days [9]. At grass root level these are being implemented by Auxiliary Nurse Midwife (ANM) for every 5,000 rural populations free of cost [10]. Community health volunteers called Accredited Social Health Activists (ASHAs), for every 1000 rural populations, have been engaged under the mission to facilitate the ANM to render maternal and child health services and establish a link between the community and the healthcare system [11].

Reasons for the program failure are many, but the major being not addressing the behavioral issues of PW and their families. Pregnancy and childbirth are unique life events and cannot be reduced to primarily biological events since the social and cultural contexts are central to the subjective and collective experiences of women [12]. Women’s age, literacy, social economic status, ethnic background, religion and culture do have a significant influence on the experiences and outcome of pregnancy. Although it is a physiological process, but it is a time when the nutritional needs of the mother and the fetus must be met through careful choice of foods. There are some avoidance and restrictions which exist in India as well as in other developing countries worldwide, which can be harmful for maternal health [13]. Behavior Change Communication (BCC) plays a decisive role in modifying such socio-cultural norms affecting the perception and nutritional practices during pregnancy.

Any public health program can enhance its chances of effectively motivating and facilitating changes in health related practices by addressing the behavioral issues and including the groups who will be most involved in the program in testing and defining the practices to be recommended [14].

With this backdrop, this study was planned involving PW and her family members to improve her nutritional status through ‘Trials of Improved Practices’ (TIPs). TIPs is developed by Manoff group, consists of a series of visits in which the interviewer and the participant analyze current practices, discuss what could be improved, and together reach an agreement on one or a few solutions to try over a trial period; and then assess the trial experience together at the end of the trial period [14].

### Objective

To examine the effectiveness of TIPs on dietary and iron-folate tablet intake during pregnancy in a rural area of Varanasi, India.

## Method

### Study setting and period

This study was conducted in *Chiraigaon* Community Development Block (CBD) of Varanasi, Uttar Pradesh state, India. Often described as the ‘heartland’ of India, Uttar Pradesh is the country’s most populous state and also has the largest rural population in India.

Varanasi district is located in the eastern end of Uttar Pradesh. It has a population of 36,82,194 and a population density of 2,399 per square kilometer [15]. The rural part of Varanasi district consists of eight CBDs [16]. CBD is a rural area allocated for administration and development in India, and covers several gram *panchayats* and local administrative units at the village level. Apart from agriculture, people are engaged in sari-weaving. Despite the soil being fertile and a traditional arts and crafts industry being present, people are poor, as they are mostly laborers, who work for low wages on the field or at the looms for a few well-to-do people. The block-level Primary Health Centre (PHC) of *Chiraigaon* is situated at *Bariasanpur* village. Apart from this, there are four additional PHCs and 30 sub-centers that render maternal and child healthcare services. The study began in May 2010 and participant recruitment in August 2010. End line assessment, after 12 weeks of the intervention, was completed in April 2011.

### Study design, participants and sample size

Quasi-experimental study design with a control group was adopted to accomplish the study objective. Four villages (Narayanpur, Rustampur, Barai and Bariyasanpur) in *Chiraigaon* CBD were selected for this study. The total population of each selected village varied from 3,000 to 4,000 with similar socio-demographic profile. A door-to-door survey was carried out in all the four villages to enlist and locate the PW. Pregnancy registers of grassroots-level health workers [Accredited Social Health Activist (ASHA) and Auxiliary Nurse Wife (ANM)] were also used to identify the cases that were missed. Assuming an absolute higher compliance of 25% for IFA supplementation in TIPs group vs. control (9%), with a two sided alpha (α) value of 0.05, power of 80% and 1:1 allocation in study groups, a sample size of 39 in each group was estimated [17]. A final sample size of 43 in each group was decided with due consideration of 10% attrition.

#### Inclusion and exclusion criteria

PW was defined as a woman of reproductive age (15–45 years) with a history of amenorrhea and positive urine pregnancy test. In the first trimester, weight gain is minimal and IFA tablet intake is avoided, for otherwise healthy PW, owing to exacerbation of nausea and vomiting. In the third trimester, PW in the study area go to their mother’s house as cultural ritual and a minimum period of 12 weeks was fixed for trial period. With due consideration of these, only PW in 13 to 28 weeks of gestation and willing to participate in the study were included. PW with acute illness, severe medical or obstetrical complications, multiple pregnancy, gestational diabetes or not staying for a minimum period of 12 weeks in the study area were excluded.

After enumerating the eligible PW (13 to 28 weeks of gestation), villages were then allocated to intervention and control groups (two villages each) by simple random sampling. Thus *Narayanpur* and *Rustampur* were selected for the implementation of TIPs on PW and remaining two villages (*Barai* and *Bariyasanpur*) were taken as controls. TIPs were applied in the intervention group through 3 home (assessment, negotiation and evaluation) visits. Written informed consent in Hindi language was taken from all the study participants before the data collection.

### TIPs intervention

Assessment visit: Interviews of PW and her family members were conducted using a pretested structured schedule and direct observations were made to understand the perception and practices of dietary and IFA intake in pregnancy. During the interview, hemoglobin (by Hemochroma plus hemoglobinometer) [18], dietary intake and weight of the PW were recorded, after taking the informed consent. As per the WHO criteria, PW were categorised as normal (≥11g/dl), mild (10–10.9g/dl), moderate (7–9.9g/dl) and severely (<7g/dl) enemies based on her hemoglobin level. [19, 20]

Negotiation visit: TIPs communication and counselling guide and home based reminder materials, with appropriate messages and pictures, were prepared by analyzing the data from assessment visit. PW were asked to select and try new recommended practices over an arbitrary period of 12 weeks. Home based reminder material was affixed in an appropriate place at home for visual reinforcement.

Evaluation visit: An evaluation was done at the end of the trial period of 12weeks to assess whether the PW could implement new practices or not. The motivating factors and barriers for the new practices were explored. Hemoglobin, dietary intake and weight of PW were recorded once again.

Only Assessment and Evaluation visits were done in control villages (*Barai and Bariyasanpur*). Hemoglobin, dietary intake and weight of the PW were recorded, after taking informed consent. At the end of the trial period, the effect of TIPs was assessed by comparing changes in weight, hemoglobin, the prevalence of anemia, compliance for IFA and dietary intake in both the study groups.

#### Statistical analysis

Data was analyzed using Statistical Package for Social the Sciences (SPSS) for Windows, Version 16.0. Chicago, SPSS Inc. Results were expressed as frequencies and proportions for categorical variables and mean and standard deviations for continuous variables. Chi square, Fisher’s exact, paired and unpaired t tests were applied to assess the significant differences in socio-demographic, obstetric and outcome measures within and across the study groups. A two sided p value of <0.05 was considered as statistically significant. Hemoglobin%, anemia prevalence, weight gain, compliance for iron-folate supplementation and dietary intake of calorie, protein, calcium and iron (by 24 hour recall method) were the key outcome measures.

## Ethical approval

Approval of institutional review board and ethics committee of Banaras Hindu University was obtained before the inception of the study (S1 and S2 Files). Informed written consent was taken from all the study participants for voluntary participation in Hindi (national language). This study is registered in Clinical Trial Registry of India (http://ctri.nic.in) and the registration number is CTRI/2015/02/005517. It was registered retrospectively after the study completion, as authors thought that the registration for ‘behaviour change intervention’ is not mandatory. The authors confirm that all ongoing and related trials for this intervention are registered. The complete study protocol can be accessed on http://ctri.nic.in/Clinicaltrials/showallp.php?mid1=10861&EncHid=&userName=TIPs

## Results

**Fig 1:**
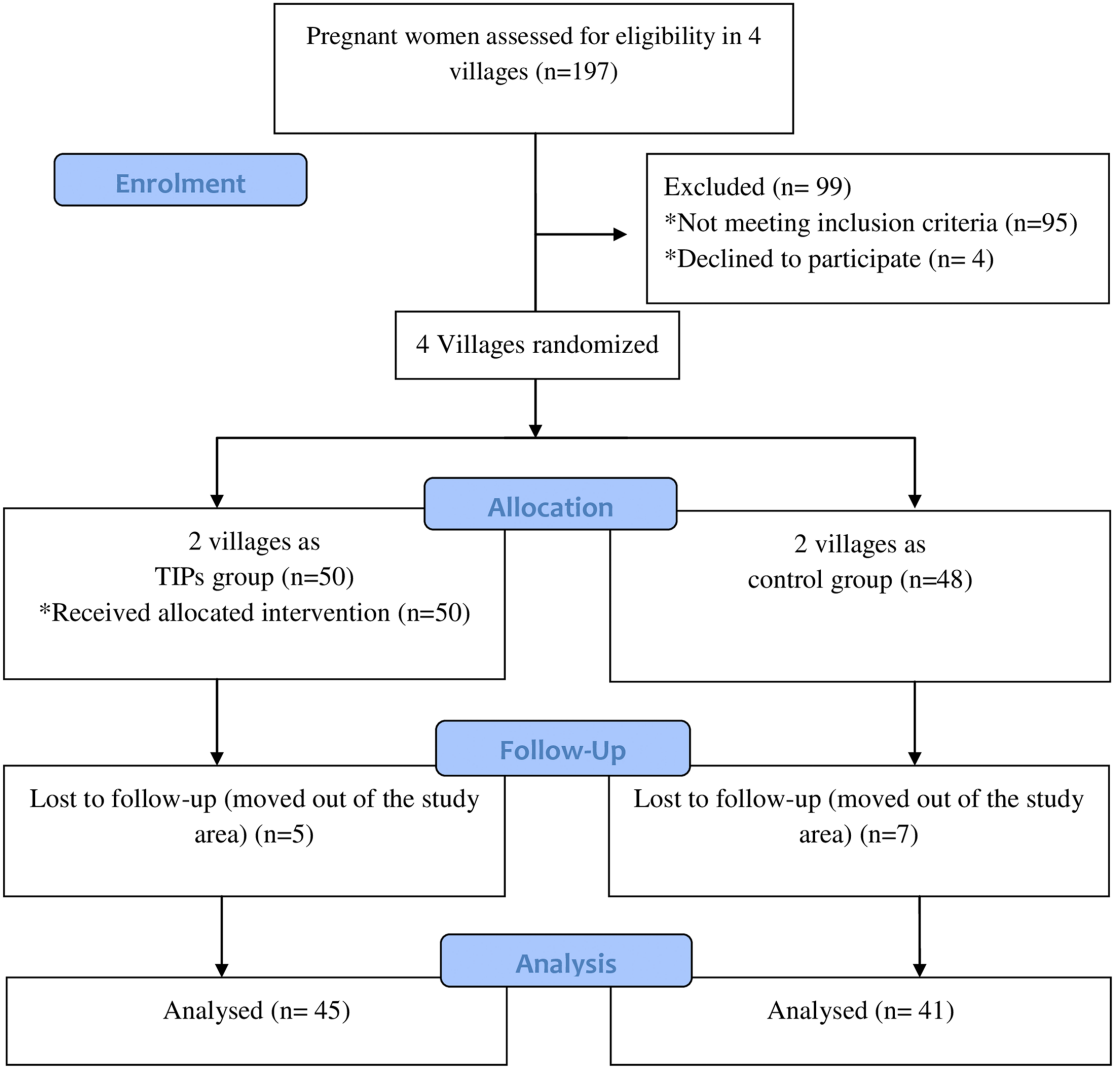
CONSORT flow diagram of the study participants.

A total of 197 PW were assessed for the eligibility in 4 study villages. Out of 197 PW, 98 eligible PW enrolled for the study. Out of 98 study participants, 50 were in the intervention group and 48 were in the control group. Five PW from the intervention and 7 from the control groups were lost during follow up. So, the overall attrition rate was 12.24% (10% in the intervention and 14.5% in control groups). Attritions were excluded during analysis (Fig 1).

The majority (62.8%) of the PW were in the age group of 21–25 years. Their mean ages were 23.18±2.82 and 22.93±3.53 years in TIPs and control groups, respectively. Nearly 90% of the PW were housewives. The joint family system was in vogue among the study groups. More than one fourth (28%) of the PW were economically below the poverty line and half of them were extremely poor(Table 1). Three out of every ten PW (28.9% in the intervention and 31.7% in control groups) were primigrvidae(Table 2). More than eighty percent of the PW had registered their pregnancy. The public health sector was the major source of antenatal care in both the study groups. As many as 84% of the PW had received or purchased IFA tablets, however, only half of them were advised about dietary intake by the health worker or doctor.

**Table 1:** Socio-demographic characteristics of the study participants (n = 86).

	Study population						
Parameter	Intervention group (n = 45)		Control group (n = 41)		Total (n = 86)		P
	No.	%	No.	%	No.	%	
**Age Group (years)**							
≤ 20	7	15.6	11	26.8	18	20.9	0.436
21–25	30	66.7	24	58.5	54	62.8	
> 25	8	17.8	6	14.6	14	16.3	
**Occupation of the pregnant women**							
Unemployed	40	88.9	35	85.4	77	89.5	0.625
Employed	5	11.1	6	14.6	9	10.5	
**Type of family**							
Nuclear	10	22.2	11	26.8	21	24.4	0.619
Joint	35	77.8	30	73.2	65	75.6	
**Economic status by type of ration card possessed**							
APL^†^	27	65.9	28	62.2	55	64.0	0.726
BPL^‡^	4	9.8	8	17.8	12	14.0	
Very poor	6	14.6	6	13.3	12	14.0	
NO card	4	9.8	3	6.7	7	8.1	
**Caste category**							
SC^§^	19	42.2	14	34.1	33	32.4	0.687
OBC^¶^	19	42.2	21	51.2	40	46.4	
Others	7	15.6	6	14.6	13	15.1	

^†^Above poverty line,

^‡^Below poverty line,

^§^Scheduled caste,

^¶^Other backward caste.

**Table 2:** Present pregnancy details and the quality of antenatal services availed by the study participants (n = 86).

Parameter	Intervention group (n = 45)		Control group (n = 41)		Total (N = 86)		P
	No.	%	No.	%	No.	%	
**Gravidity**							
1	13	28.9	13	31.7	26	30.2	0.804
2	25	55.6	20	48.8	45	52.3	
≥3	7	15.6	8	19.5	15	17.4	
**Duration of pregnancy (completed months)**							
3	13	28.9	9	22.0	22	25.6	0.473
4	13	28.9	8	19.5	21	24.4	
5	10	22.2	11	26.8	21	24.4	
6	9	20.0	13	31.7	22	25.6	
**Source of antenatal care services**							
Public sector	35	77.8	31	75.6	66	76.7	0.629 ^‡^
Private sector	3	6.7	2	4.9	5	5.8	
Not availed	7	15.6	8	19.5	15	17.4	
**Antenatal care services availed**							
TT^§^ shot/s	34	89.5	30	90.9	64	90.1	0.800
Received IFA^†^	32	84.2	28	84.8	60	84.5	0.776
Dietary advice	20	52.6	17	51.5	37	52.1	0.780

^†^Iron Folate tablet;

^‡^computed by clubbing public and private sectors;

^§^TT: Tetanus Toxoid

In this study as much as 60% and 73% of the PW in the intervention and control groups had suffered from one or the other pregnancy related ailment. Nearly half of the study subjects in both the groups told that ailments resolved spontaneously. However, one tenth of them had to consult a doctor. Forgetfulness and initial side effects of IFA tablets (viz. nausea, vomiting, bad taste or smell and giddiness) were the major barriers affecting the compliance in both the study groups. Non-availability of IFA tablet was the issue, according to 10% of the PW.

The majority (75.6% in the intervention and 78% in control groups) of the study participants were vegetarians. More than half (55.8%) of the PW in both the study groups had decreased their dietary intake since conception. Many of the non-vegetarian PW had stopped non vegetarian intake since conception and similarly vegan PW had reduced intake of protein rich pulses. This was due to the prevailing dietary misconceptions such as fear of the big or black baby, overeating is harmful etc. among them. The concept of hot and cold foods was in vogue among the PW. Egg, turmeric and tea were considered as ‘hot’ and were avoided in pregnancy. According to 80% of them, aroma of the pulses while cooking was nauseating and repulsive. Many (52.1%) of them were unaware of additional dietary demand during gestation and lactation. Even most of the mother-in-laws and husbands stated that loss of appetite is common and is a normal phenomenon during pregnancy.

**Fig 2:**
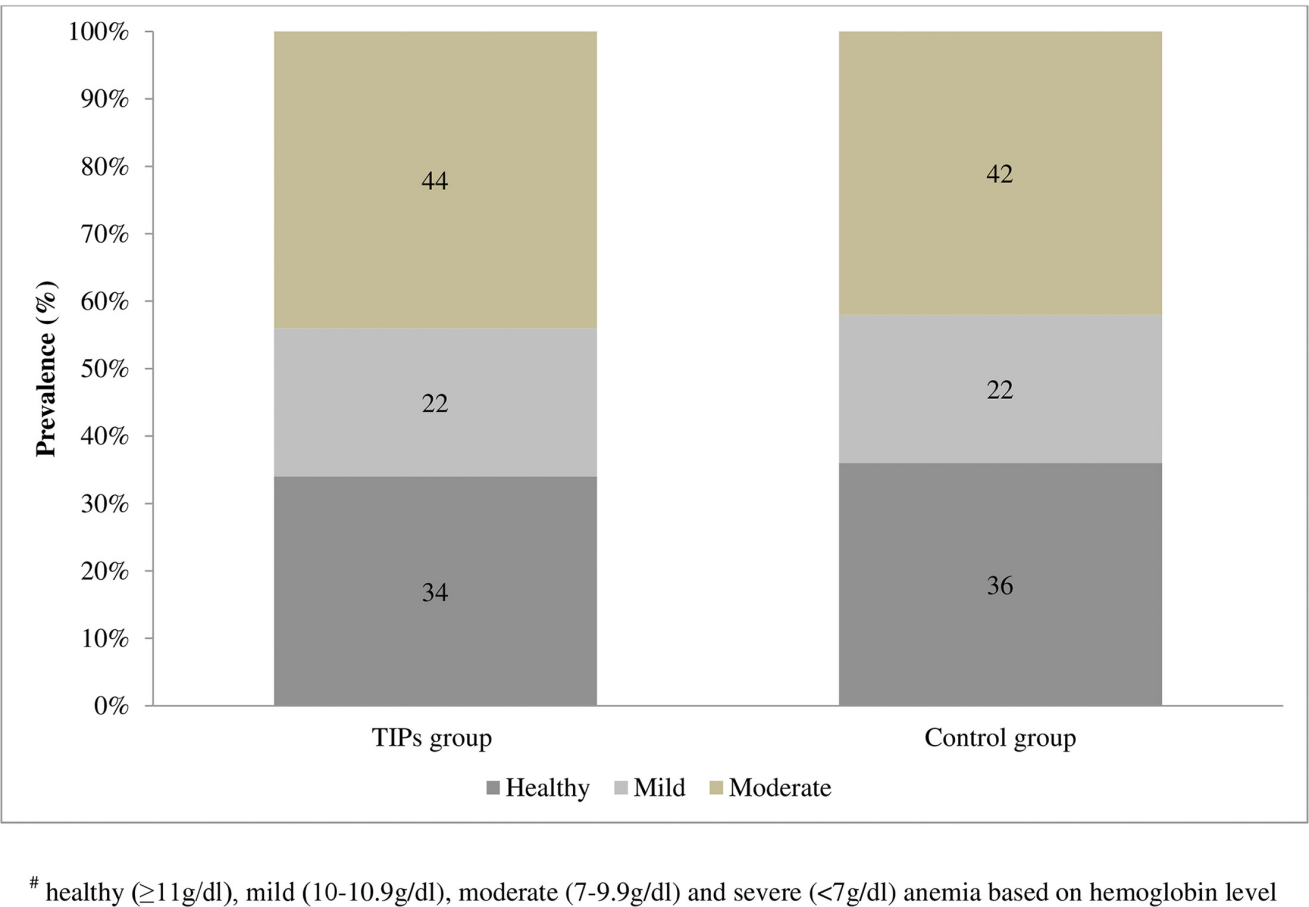
Percentage prevalence of anemia in both the study groups before the TIPs intervention. Hemoglobin level cut-offs: healthy (≥11g/dl), mild (10–10.9g/dl), moderate (7–9.9g/dl) and severe (<7g/dl) anemia

Before the TIPs intervention mean hemoglobin levels were 10.34 ± 1.56 g/dl and 10.15 ± 1.59 g/dl in the intervention and control groups, respectively(Table 3). As much as 65.9% (95% CI: 52.1–79.8%) and 64.4% (95% CI: 49.7–79.1%) of the PW in the intervention and control groups were anemic, respectively (Fig 2). And the overall prevalence of anemia (as per WHO criteria) in the study population was 65.1% (95% CI: 55.03–75.17%). Most of the PW were moderately anemic in both the study groups. More than three fourths of the PW were weighing less than that of an Indian reference woman (55 kg) [21]. The observed differences in mean hemoglobin (p = 0.577), the prevalence of anemia (p = 0.823), body weight (p = 0.181) and dietary nutrient intake (calorie, protein, iron and calcium) between both the study groups were not statistically significant (p>0.05). Both the study groups did not differ significantly (p>0.05) in their socio-demographic profile, obstetrical variables and the quality of antenatal services received.

**Table 3:** Hemoglobin level and weight of the pregnant women in both the study groups before and after the intervention (n = 86).

	Study population (n = 86)			
Parameter	Intervention group (n = 45)	Control group (n = 41)	t	P
	Mean ± SD	Mean ± SD		
**Before intervention**				
Hb^†^ (g/dl)	10.34 ± 1.56	10.15 ± 1.59	0.556	0.577
Weight (Kg)	48.64 ± 8.23	46.68 ± 5.01	1.319	0.181
**After intervention**				
Hb^†^ (g/dl)	11.5 ± 1.24	10.4 ± 1.38	4.018	<0.001^§^
Weight (Kg)	50.4 ± 8.49	46.9 ± 5.53	1.319	0.027^¶^
**Change in Hemoglobin and Weight**				
Hb^†^ (g/dl)	1.16 ± 1.41	0.21 ± 1.089	3.47	<0.001^§^
Weight^‡^	326.9 ± 91.8	244.6 ± 97.4	4.033	<0.001^§^

^†^Hemoglobin,

^‡^weight change: grams per week

^§^Highly significant

^¶^Significant

**Fig 3:**
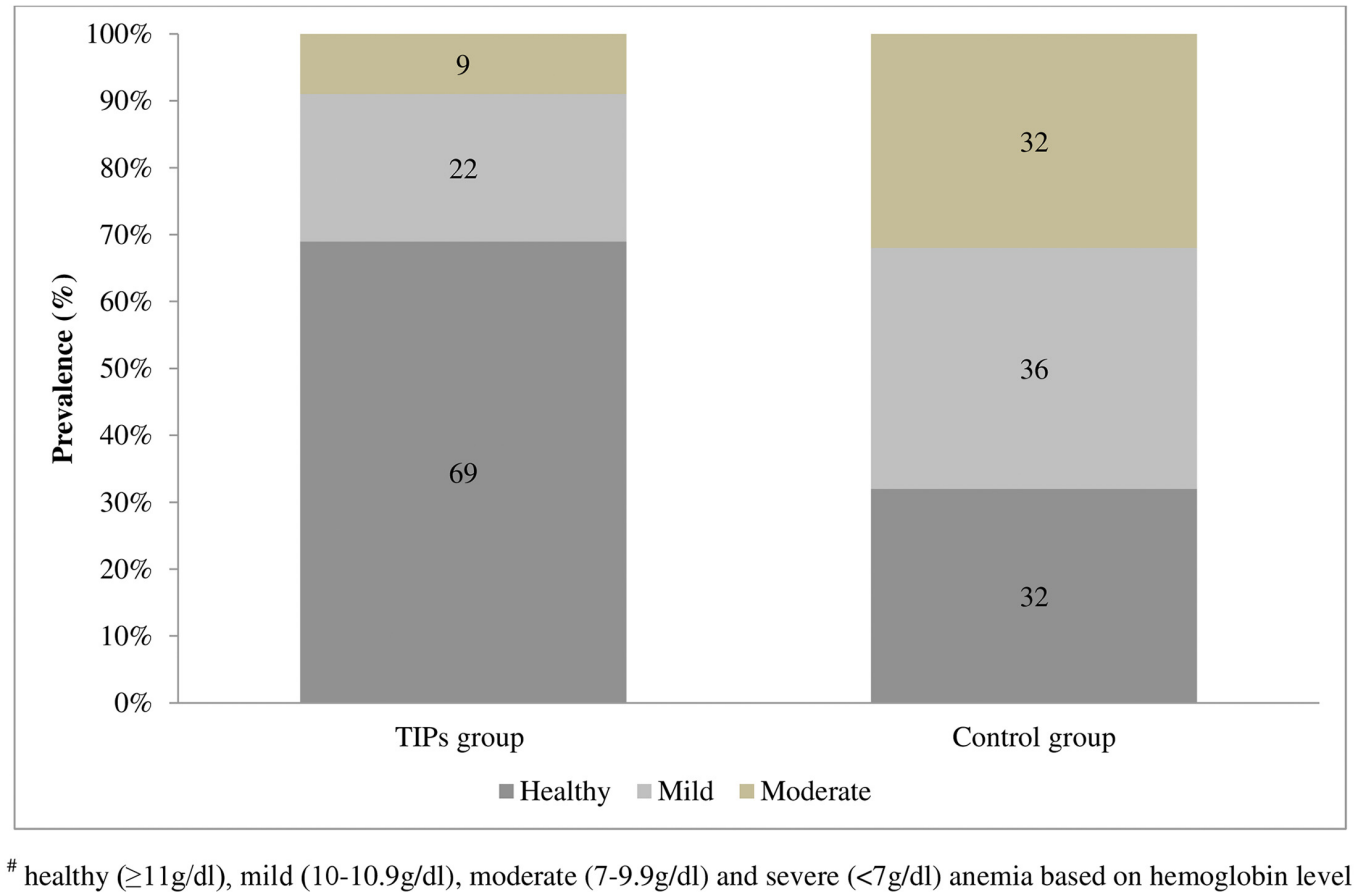
Percentage prevalence of anemia in both the study groups after the TIPs intervention. Hemoglobin level cut-offs: healthy (≥11g/dl), mild (10–10.9g/dl), moderate (7–9.9g/dl) and severe (<7g/dl) anemia

At the end of the trial period, mean hemoglobin were 11.5±1.24 g/dl and 10.37 ±1.38 g/dl in the intervention and control groups, respectively (Table 3). The prevalence of anemia, reduced by half (65.9% to 31.1%) in the intervention group and increased by 2.4% in the control group, as compared to baseline values (Fig 3). The differences in the mean final hemoglobin (p<0.001), weight (p = 0.027) and the prevalence of anemia in both study groups were statistically significant (p = 0.001). Paired t test revealed that the TIPs group displayed significant differences in hemoglobin level [t(44) = 5.3; p<0.001] and weight [t(44) = 4.3; p<0.001]of the PW before and after the intervention. However, the observed differences in initial and final hemoglobin level [t (40) = 1.8; p = 0.08] and weight [t(40) = 0.47; p = 0.64] of the PW among the controls were statistically not significant.

Compliance for IFA was assessed by number of IFA taken by participant recalls and was verified by checking used tablet packet/s at the end. As much as 85% of the PW in TIPs group were compliant for IFA and only 38% were compliant among controls.

Mean intake of calories increased by 161 and 65 kilocalories in the intervention and control groups, respectively. (p>0.05) Mean intake of protein increased by 1.78gm in the intervention group on the contrary, it was decreased by 1.81gm in the control group. The mean protein intakes of study groups differed significantly at the end of the trial period (p = 0.003) (Table 4). More than two thirds of the PW in the intervention group were taking one extra meal and only one third of those in the control group were doing the same.

**Table 4:** Dietary intake of calorie, protein, Iron and Calcium by 24 hour recall method in both the study groups before and after the intervention (N = 86).

	Study population (N = 86)			
Dietary intake	Intervention group (n = 45)	Control group (n = 41)	t	p
	Mean ± SD	Mean ± SD		
**Before intervention**				
Calorie (kcal)	1686.6 ± 275.6	1731 ± 298.1	0.718	0.475
Protein (gm)	53.68 ± 20.24	47.18 ± 8.77	1.898	0.06
Iron (mg)	19 ± 7.02	19.05 ± 6.63	-0.03	0.973
Calcium (mg)	366.2 ± 211.1	357.24 ± 258.7	0.177	0.860
**After intervention**				
Calorie (kcal)	1847.1 ± 261.7	1796.1±298.8	0.844	0.401
Protein (gm)	55.46 ±18.9	45.37±9.83	3.060	0.003^†^
Iron (mg)	21.58 ±7.25	19.96±6.59	1.078	0.283
Calcium (mg)	402.92 ± 204.8	345.76±236	1.201	0.232

^†^Highly Significant

Much of the TIPs group PW appreciated the interpersonal communication, affixed reminder materials and family support as motivators and sustenance of enhanced dietary and IFA intake. Explaining the benefits of additional dietary and IFA intake as ‘PW will have a healthy and intelligent baby’ was the key to enhance their acceptability and the same was visually reinforced by the reminder material. Similarly, misconception about the egg was clarified and its necessity for the baby’s growth was elucidated. Milk was advised for those who avoid tea. The advice of topping the dhal with chutney, pickle and/or green leafy vegetables could help them overcome the aroma induced nausea.

## Discussion

### Main Findings

At the end of the trial period, the intervention group showed a significant (p<0.05) improvement in gestational weight gain, IFA compliance, hemoglobin levels, dietary intake, and prevalence of anemia when compared to controls. Interpersonal communication, active participation of family members and the process of affixing reminder materials facilitated the PW to overcome the barriers of dietary intake and enhanced the IFA compliance. Not just the quantity, but the quality of the diet, too, improved in terms of increased consumption of green leafy vegetables and locally available pulses by PW.

### Interpretation

Immense physiological changes take place in the woman’s body during pregnancy. All these changes demand an alteration in the woman’s lifestyle, including her dietary habits, to cope up with the increased requirement of nutrients. Various studies indicate that diet of PW in India is generally deficient, especially so in the rural areas and in the urban lower class [22–25]. Poor compliance with IFA tablets is a well known phenomenon among PW. During pregnancy, women’s access to foods is even more restricted in the traditional Indian household through taboos and ritual observances, which are widely documented in both rural and tribal populations.

This interventional study was carried out to assess the effect TIPs on nutritional status of pregnant women. Owing to profound hormonal changes and minor ailments (nausea, vomiting and morning sickness), a loss of appetite would be expected in the first trimester. Although this effect gets blunted as the pregnancy advances, most of the PW continue to consume a deficient diet because of the myths and taboos, unless motivated. This was reflected in this study by a relatively higher prevalence of anemia and reduced dietary intake since conception, in both study groups. Shahid A et al., [13] Ahlquist M et al., [26] Grewal SK et al. [27] and Nag et al. [28], in their studies conducted in different countries, also reported decreased dietary intake among PW.

Interpersonal communication, endorsing the active participation of family members and home-based reminder materials with encouraging messages and pictures for visual reinforcement, were effective TIPs strategies to improve the nutrition status of the PW. At the end of the trial period, the intervention group showed a significant quantitative and qualitative enhancement in dietary intake and IFA compliance, as compared to controls. Active participation of husbands and their mothers-in-law boosted PWs’ confidence and also ensured sustainability during the trial period. Previous studies also recommended the involvement of the partner and decision-maker in antenatal care, as this would result in greater net impact on maternal health behaviors, compared to educating the women alone [29–31].

Any chronic disease demanding long term treatment poses the problem of patient compliance for medication/s. Poor adherence/compliance is attributed as a primary cause for failure to achieve expected full treatment benefits [32,33]. In this study, forgetfulness and initial side effects were the major hurdles in IFA tablet intake. This is corroborated by the findings of Oriji VK et al [34] and Seck BC et al [35]. The reminder materials used in this study could successfully address these issues through visual reinforcement. An important implication of this study is the feasibility of TIPs so that the same can be emulated by grass root level health care workers like ASHA and ANM. ASHA or ANM is expected to pay at least 1 home visit, apart from the 4 regular ANCs at the healthcare set up, during pregnancy and to educate the PW and their family members about various aspects of antenatal and nutritional care. This home visit has the maximum impact if utilized properly.

The same concept was used in this study through TIPs. PW and their family members were educated about the antenatal care with a special emphasis on diet and IFA with the help of the reminder materials and the same was affixed in her house in an appropriate place for visual reinforcement. As the study progressed, we could do both the assessment and the negotiation in one visit. Thereafter, the final assessment was done at the end of the trial period. This was essential because for ANM/ASHA practically it was not possible to make more than one home visit. We purposefully did not visit during the trial period to crosscheck IFA and dietary compliance to avoid the Hawthorne effect (i.e. The improved performance of the participant on continuous or frequent observations by the investigator) [36,37].

Many strategies like reminder medicine blister packs, personalized or automated telephonic call or short message service reminders have been proven to be effective in enhancing the adherence in various diseases [38–42]. Use of reminder material is more feasible and effective in Indian rural settings. Based on the findings of the present study, one of the pages of maternal and child protection booklet, displaying key messages with pictures, supplied under RMNCH+A program could be affixed in an apt place of PW’s house as a reminder material [43]. Large scale multi-centric cluster randomized trials are needed to assess the external validity of this.

### Strengths and Limitations

This study emphasizes TIPs as an effective tool to understand the local beliefs, perception and practices of nutrition in pregnancy for developing culturally acceptable and effective BCC material. TIPs method is feasible and could be easily emulated by grass root level workers like ANM and ASHA.

Behavior change is a long term process requiring continues efforts. We did not follow the PW till the delivery or in lactation period to check their compliance for the adopted behavioral changes. Hence, sustainability of the observed outcomes in this study is questionable. Considering the vast regional socio-cultural diversity affecting the nutritional perceptions and practices during pregnancy in India and relatively small sample studied, findings may not be applicable to other populations.

## Conclusion

TIPs found to be an effective approach to improve the nutrition status of PW in the study area. Interpersonal communication, endorsing the active participation of family members and home based reminder materials, with encouraging messages and pictures for visual reinforcement, were the effective TIPs based strategies to improve the nutritional status of PW. The effect of TIPs could be further explored on larger sample representing different socio-cultural and geographical areas.

## Supporting Information

S1 File Study trial protocol. (PDF)

S2 File Approval letter of the institutional ethics committee. (PDF)

S3 File TREND statement checklist. (DOC)

## Abbreviations

ASHA: Accredited Social Health Activist
ANM: Auxiliary Nurse Midwife
BCC: Behavior Change Communication
CBD: Community Development Block
IFA: Iron Folate
PW: Pregnant Woman/en
SPSS: Statistical Package for Social Sciences
TIPs: Trials of Improved Practices
