# Supplementary material for: Trials of Improved Practices (TIPs) to Enhance the Dietary and Iron-Folate Intake during Pregnancy- A Quasi Experimental Study among Rural Pregnant Women of Varanasi, India
Source: PLoS One. 2015 Sep 14;10(9):e0137735. doi: 10.1371/journal.pone.0137735 (PMC4569533; doi:10.1371/journal.pone.0137735)
Supplement: S1 File — (PDF) [file pone.0137735.s001.pdf]

SYNOPSIS FOR REGISTRATION OF TITLE OF THESIS FOR M.D. (COMMUNITY MEDICINE)

1.	(a) Name of the candidate	:	***Dr. Siddharudha Shivalli***
	(b) Name of the course	:	M.D. (Community Medicine)
	(c) Year and month of registration	:	May, 2009
	(d) Year and month of examination when due to appear	:	March, 2012
2.	Title of the proposed thesis	:	**STUDY OF EFFECTIVENESS OF TIPs (TRIALS OF IMPROVED PRACTICES) AGAINST MATERNAL ANEMIA IN CHIRAIGAON BLOCK OF VARANASI**
3.	Name and Designation of the Guide		***Dr. Ratan Kumar Srivastava***<br>Professor and Head,<br>Department of Community Medicine<br>Institute of Medical Sciences<br>Banaras Hindu University<br>Varanasi- 221 005
4.	Name and Designation of the Co-Guide		***Dr. G.P. Singh***<br>Assistant Professor(biostatistics), Department of Community Medicine, Institute of Medical Sciences,<br>Banaras Hindu University<br>Varanasi- 221 005
5.	(a) Department where thesis work will be carried out	:	Department of Community Medicine<br>Institute of Medical Sciences<br>Banaras Hindu University<br>Varanasi- 221 005
	(b) Which other department of the Institute/University will co-operate in the proposed thesis	:	NONE

6. STATE BRIEFLY OBJECTS AND SCOPE OF THE PROPOSED RESEARCH WORK:

Prophylactic 100 IFA tablets intake by all pregnant women would drastically reduce anemia related complications of pregnancy. Though the concept is simple and straightforward, its implementation has failed to an unexpected level in most of the developing countries including India. In India, **National Anemia Prophylaxis Programme** started in 1970 promoting 100 IFA tablets (60 mg elemental Iron and 0.5 mg folic acid) intake by all pregnant women. From time to time the programme underwent modifications like increasing the dose of elemental Iron to 100 mg and renamed as **National Anemia Control Programme** (1991) and **Maatri Suraksha Abhiyan**. Later it was taken-up by **Maternal Child Health** Dept. (part of Ministry of Health and Family Welfare), now it's being implemented as a part of **Reproductive Child Health (RCH)** Programme.

Although anemia control programmes are running since 1970, anemia still continues to be a major public health problem in India and one of the leading causes of maternal deaths. According to NFHS-3, 59% of pregnant women, 58% of lactating mothers and 46% of highest wealth group pregnant women are anemic, major reasons being increased demand, poor Iron intake and improper dietary practices decreasing absorption of Iron and poor compliance for 100 IFA tablets intake, during pregnancy. Three fourths of pregnant women receive any antenatal care, 65% initiated IFA intake (given/purchased) but only 23% took for 90 days or more.

Situation is still worse in Utter Pradesh which accounts for highest number of maternal deaths and MMR in India (440/1000 live births, SRS-2004-06). More than 50% pregnant women are anemic, 26.3% have had 3+ antenatal check-ups and only 8.7% took 90/more IFA tablets indicating huge gaps in both expected number of pregnant women to receive antenatal care and completing IFA intake. Every day 68 women die of pregnancy related complications in which 14 are due to anemia, in Utter Pradesh. Therefore **better compliance for minimum 100 IFA tablets intake and proper and adequate dietary practices are the issues in focus for the survival of pregnant women.**

Iron is the most studied micronutrient but we have least consensus on it and pessimism is prevailing among policy makers and workers involved in Iron related programmes. Reasons for failure are many but the major being not addressing the behavioral issues of pregnant women and their families. Every public health program must address to **"behavioural issues"** if it hopes to reach its desired impact. Any public health program can enhance its chances of effectively motivating and facilitating changes in health-related practices by including the groups who will be most involved (pregnant women) in the program in testing and defining the practices to be recommended.

So there is a need of research involving pregnant women and their families to understand their perception regarding antenatal care, IFA tablets intake and dietary practices during pregnancy, so as to identify socio-cultural, demographic and other barriers for complete antenatal care. And finally bringing change in the behaviours to overcome the barriers by negotiation with pregnant women and their families.

TIPs (Trials of Improved Practices)is one of the behaviour changing ,formative research techniques, focuses on behaviour, what people do, rather than knowledge, or what people know or believe. Trials are the best way to gauge the acceptability of proposed new practices and learn how to promote and support them.

7. Objectives of proposed research work:

To examine the effectiveness of 'Trials of Improved Practices' (TIPs) method of behavior change communication on dietary and iron-folate intake during pregnancy

8. Hypothesis of the proposed research work:

TIPs would improve the compliance for 100 IFA tablets intake and dietary intake by pregnant women and consequently their anemic status.

9. Scope of the study:

- Study would focus on socio-cultural and demographic factors affecting the compliance and dietary practices of pregnant women.
- From this study we would come up with new/modified suggestions which could be implemented at community level via ASHA (Accredited Social Health Activist) and other grass root workers to improve anemic status and its complications in pregnant women, in eastern U.P.

10. LACUNAE IN KNOWLEDGE ON THE SUBJECT

Sufficient literature is not available on current dietary practices and patterns of behaviors of pregnant women for IFA intake in the study area. Ongoing reproductive and child health programmes have failed to create an impact in maternal anemia at community level.

11. PRELIMINARY WORK THE CANDIDATE HAS ALREADY DONE ON THE PROBLEM

NONE

12. BRIEF ABOUT ANY WORK PERTAINING TO THE PROPOSED STUDY THAT HAS BEEN DONE IN THE DEPARTMENT EARLIER

A study comparing the oral iron therapy with parenteral route for the treatment of maternal anemia has been done. However it doesn't address the compliance of oral iron therapy. It does not address the dietary practices or compliance of oral therapy.

Recently 2 projects on maternal anemia have been taken-up in the department. However projects deal with improving the skills of Mukhya sevikas and AWW in implementing maternal anemia reduction programme and assisting demand generation activity in the community through mass communication and social mobilization. Projects neither focus on reasons for poor compliance for 100 IFA tablets nor involve in any kind of interaction and feedback from pregnant women.

REVIEW OF LITERATURE

TIPs (Trials of Improved Practices)

Trials of Improved Practices (TIPs) are a formative research technique developed by the Manoff Group. Using TIPs, program planners select and pre-test the actual practices that a program will promote. In essence the procedure consists of a series of visits in which the interviewer and the participant analyze current practices, discuss what could be improved, and together reach an agreement on one or a few solutions to try over a trial period; and then assess the trial experience together at the end of the trial period. The results are moved directly into program design.

TIPs focuses on behaviour, what people do, rather than knowledge, or what people know or believe. Trials are the best way to gauge the acceptability of proposed new practices and learn how to promote and support them. TIPs evolved out of commercial marketing and anthropology research methods. Its objective is to define feasible and efficacious behaviours and learn whether they are also acceptable and feasible. The actual technique of TIPs combines the **"advertising-design concept"** of **"concept testing"** (Market Navigation, Inc.) with product testing in order to modify the practice or product before it is introduced into the market, based on feedback from a small sample whose members actually try using the product in their daily lives.

Through TIPs, planners learn from families, providers or communities:

- what practices the program should promote, eliminate or modify;
- what are the most effective motivations and most significant barriers to new practices;
- what level of change in particular behaviours the program can expect; and in some cases,

- what level of health or nutrition impact the program can expect.

The first use of TIPs (at the time called concept or intervention testing) was by Manoff International in the 'Nutrition Communication/ Behavior Change project' in Indonesia in the late 1970s. This first use of trials proved to be extremely helpful in program design. Planners learned that it was feasible to promote breastfeeding equally from both breasts (rather than the customary one); how to improve instructions for preparing homemade oral re-hydration solution; and of the need to modify weaning food suggestions in different regions. Mothers themselves came up with suggestions on ingredients and cooking technique that were incorporated into program message.

Since this first experience, TIPs have been effectively used in formative research related to infant, child, and maternal nutrition in many countries.Over the past decade, TIPs have been applied to other public health issues including HIV/AIDS, school health , infectious disease control, maternal health and family planning. WHO recommends using a simplified version of TIPs to adapt the generic "mothers card" that is part of IMCI counseling to reflect specific foods and other behaviors appropriate for each country or region of a country.

Some Recent Applications of the TIPs Methodology

Kentucky, Mississippi, California	Healthy lifestyles for primary school-age children	Multiple behaviours related to physical activity, eating and drinking	2006
Mozambique and Zambia	Injection safety practices	Multiple behaviours with different providers related to prescribing, injecting and waste disposal	2004
Dominican Republic, Nicaragua, Peru	Basic hygiene (hand washing, consuming clean water, safe feces disposal)	Individual and family practices in home and environment, also purchasing subsidized essential hygiene products	2001-2003
Dominican Republic, El Salvador (CHANGE Project)	Dengue	Family actions to avoid mosquito breeding in household water containers	2001-2003
South Africa	Indoor air pollution	Repairing stoves, improving ventilation, shortening burning time, keeping young children away from smoke	2002

Dominican Republic<br>Every public	Young child feeding	Focus on calories and nutrient content in normal<br>times and when children are sick or recovering	2001
Pakistan	Men's role in family planning<br>decision making	Discussing with spouse, learning about methods,<br>making joint decision, starting a modern method	2000
Zambia	Insecticide-treated bed nets	Obtaining and appropriate use of treated bed nets	1997

What behaviours can be tested?

Most health behaviours can be tested in TIPs. There are some limitations, however. The following types of behaviours are more difficult, or even impossible, to test.

- Behaviours that stretch over long time periods (e.g., get your child fully immunized by age 1,breastfeed your child for at least 2 years)
- Behaviours that are appropriate only at rare or unpredictable times (e.g., appropriate care seeking for obstetrical emergencies; communities helping with emergency transport during emergencies)
- Behaviours with major external barriers such as poor policies
- Behaviours that require collaboration or approval of many colleagues or Supervisors

Behavioural changes that are difficult or not possible to test in TIPs can be explored through other methods such as in-depth interviews or focus group discussions.

TIPs METHODOLOGY:

TIPs are the second phase of the formative research process. The first phase consists of a **literature search, expert interviews and (often) in-depth interviews and observations** (with the key participant groups). Based on the results, the research team designs the TIPs - designates the types of participants and the sampling plan and develops counselling and motivation guides for the problem practices of interest in health and nutrition.

Field Work

It usually consists of two or three visits. In the first (assessment) visit, the families' situation is analyzed through interview questions and sometimes through a food frequency assessment or dietary recall. Bring back the interview information and have the research team analyze the findings and assess the problems and best menu of behavioral solutions.

Negotiation

In the negotiation visit, the researcher gives feedback to the pregnant women (or others) on their practices (both on what they are doing well and areas they might improve) and gives several relevant suggestions of actions the pregnant women might try for a trial period. (This period is often 5-7 days but may be as long as a few months.) These suggestions are discussed thoroughly and the pregnant women selects one to three of these ideas for trial. In

the final (evaluation) visit, the interviewer learns what the pregnant women did, how and why; how she felt about the trial experience; what was easy and difficult; if she discussed the new behaviours with anyone and what they said; how she would recommend the same practice to a friend, etc. If it was part of the initial visit, a food frequency assessment or dietary recall will be repeated.

Analysis

Analysis of TIPs findings is relatively straightforward and generally easier than analyzing in-depth interviews or focus group discussions. The analysis is both quantitative and qualitative. For example, how many pregnant women had a particular problem, how many accepted the improved behaviours and which behaviours did they select, what were the most effective motivators, what was the pregnant womens' experience and success during the trial, what modifications or suggestions did they make and why, who in the family and community influenced their behaviour, what were the main barriers they had to overcome and how did they do this, what were their perceived benefits, and how many intended to continue the new practice.

METHODOLOGY OF PROPOSED RESEARCH WORK

Study area: Four villages of Chiraigaon Community Development Block of Varanasi, India.

Study sample: 102 pregnant women in Chiraigaon block, Varanasi.

Sample size: Assuming an absolute 25% higher compliance for IFA supplementation in TIPs group vs. control (9%), with an α error of 0.05 and a power of 80%, a sample size of 51 participants in each group is estimated. As this a mixed (qualitative and quantitative) study with the purpose of testing a short term behaviour trial and involves one-to-one interaction and counselling with the family, large sample is neither essential nor feasible. A small sample that is representative of ethnicity and socio-economic differences of the study area, will give a valid data.(Kanani et al,1998)

Study design: Community based quasi experimental study with a control group

Study period: From May 2010 to July 2011.

Inclusion criteria: In first trimester, weight gain is minimal and IFA tablet intake is avoided owing to exacerbation of nausea and vomiting. In third trimester, PW in India go to their mother's house as cultural ritual and a minimum period of 12 weeks was fixed for trial period. With due consideration of these, only pregnant women in 13 to 28 weeks of gestation and willing to participate in the study will be included.

Exclusion criteria: Pregnant women with acute illness, severe medical or obstetrical complications, multiple pregnancy, gestational diabetes or not staying for a minimum period of 12 weeks in the study area.

After enumerating the eligible pregnant women, villages will be allocated to intervention and control groups (two villages each) by simple random sampling. TIPs method of behaviour change communication would be applied in intervention group through 3 home (assessment, negotiation and evaluation) visits. Only Assessment and Evaluation visits will be done in control villages. Written informed consent in *hindi* language was taken from all the study participants before the data collection.

TIPs intervention:

1] Assessment visit: In-depth interviews of pregnant women and their family using semi-structured questionnaire and direct observations to understand the perception and practices of IFA intake and diet in pregnancy. During interview, Hb% (Hemo Cue method), weight and height will be recorded, after taking informed consent.
2] Negotiation visit: TIPs communication and counselling guide will be prepared by analysing the data from assessment visit. Pregnant women will select and try new recommended practices over an arbitrary period of 12 weeks.
3] Evaluation visit: At the end of the trial period, evaluation will be done whether pregnant women could implement new practices or not and what were the motivating factors/barriers to implement. The pregnant womens' experience and success during the trial would be noted along with a record of modifications or suggestions they made and why, who in the family and community influenced their behaviour, what were their perceived benefits, and how many intended to continue the new practices. Hb% and weight of pregnant women will be recorded once again.

Outcome Measures: Hemoglobin%, anemia prevalence, weight gain, compliance for iron-folate supplementation and dietary intake of calorie, protein, calcium and iron (by 24 hour recall method).

Analysis: Data will be analyzed using Statistical Package for Social the Sciences (SPSS) for Windows, Version 16.0. Chicago, SPSS Inc. Results will be expressed as frequencies and proportions for categorical variables and mean and standard deviations for continuous variables. Chi square, Fisher's exact and unpaired t tests would be applied to assess the significant differences in socio-demographic, obstetric and outcome measures across the study groups. A two sided p value of <0.05 would be considered as statistically significant.

Gantt chart displaying timeline for various activities

Year →	2010												2011						
Activity	JAN	FEB	MAR	APR	MAY	JUN	JUL	AUG	SEP	OCT	NOV	DEC	JAN	FEB	MAR	APR	MAY	JUN	JUL
Literature search	■	■	■	■	■	■	■	■	■	■	■	■	■	■	■	■	■	■	■
Ethical approval			■																
TIPs phase						■	■	■	■	■	■	■	■	■	■	■			
Data analysis																	■	■	
Write-up																■	■	■	■

References:

1. The Manoff Group, 2001 Street NW, Suite 400, Washington DC 20009 www.manoffgroup.com (28-11-2009)
2. Shubhada Kanani, 1998: Behaviour change trials for better nutrition of pregnant and lactating women in Angharah block Bihar.
3. Barnes, Brendon R., Angela Mathee, Lonna B. Shafritz, Laurie Krieger,and Susan Zimicki. "A Behavioral Intervention to Reduce Child Exposure to Indoor Air Pollution: Identifying Possible Target Behaviors." Health Education and Behavior June 2004, 31(3): 306-317.
4. Barnes, B. R., Mathee, A., Krieger, L., Shafritz, L., Favin, M. and Sherburne, L.Testing selected behaviours to reduce indoor air pollution exposure in young children. Health Education Research 2004, 19(5): 543-50.
5. Centre for Health, Population, and Nutrition (CHEPON).Qualitative Research on Infant Feeding in Oyo and Osun States, Nigeria. Washington, D.C.: Wellstart International, October 1995.
6. Changing Behaviors: Guidelines on Using Research to Increase Consumption of Micronutrients. New York: Helen Keller International, 1998.(Prepared by The Manoff Group and Helen Keller International.)

(Dr.Siddharudha Shivalli)
Juniornresident, IMS, BHU, VARANASI.

CONSENT FORM

INTRODUCTION

Namaste, My name is .... , from department of community medicine, IMS, BHU, Varanasi. We are conducting a study in chiraigaon block to learn and improve dietary practices and 100 IFA intake by pregnant women (study of effectiveness of tips methodology against maternal anemia in chiraigaon block of varanasi district).Participation in this study is completely voluntary. However we would very much appreciate your participation in this study, since your views are very important.

METHOD OF THE STUDY

It consists of Focussed Group Discussions (FGD) and 3 home visits. In 1st visit (this visit), I'm going to ask you and your family members about the care, diet and 100 IFA intake during pregnancy. The questionnaire usually takes about 30-40 minutes to complete. At the end we would like to take a small drop of blood by a small prick to your **left ring finger** to measure the level of Hb% (Iron level),as its measurement is very important in pregnant women. Procedure should cause you very little discomfort. However it's completely your decision to allow us to take the sample. Result of the test will be explained within 30-40 seconds. The sample will be destroyed after the study and won't be used in any other research. In addition, we would like to take measurements of your weight and height, to assess your health and nutritional status. You are requested to participate in a FGD on the same subjects of questionnaire so that we can understand your perception and practices in a better way.

In 2nd visit we would educate you about some simple practices to improve your health status. You only will select and try out some or all of them over an arbitrary period of 12 weeks. At the end of trial period we will come for 3rd visit to ask about your experience of the trial. In addition we would like to take repeat measurements of Hb% (by a small prick to your **left ring finger**), weight and height.

POTENTIAL BENEFITS

Your participation in the study would help us to assess the present health and nutritional status of pregnant women and educate you about the same. Experience from the trial would help you to improve your health and nutritional status so that you would be able to reproduce a physically and mentally healthy child. And you would be able to share your trial experience with other pregnant women. Your feedback from trial experience would be used to disseminate healthy messages in the community for the betterment of pregnant women.

POTENTIAL DANGERS

No potential dangers are involved in this study. We will take all aseptic precautions by using disposable lancets (needles) to avoid any kind of infection.

CONFIDENTIALITY

The information concerning your participation in the study will be kept confidential to the full extent permitted by the law and used only for scientific purpose. No one except members of the research team will have access to the test results. Your name will not be used in any report or released in any way.

RIGHT TO WITHDRAW FROM THE STUDY

You have rights to refuse answering any or all questions, to undergo any test or measurements during the study. You also have the right to withdraw from the study at any time. However we hope that you will fully participate in the study, as your views are important.

Please let me know if anything I've stated isn't clear and I'll be happy to explain it further to ensure you understand.

INFORMED CONSENT OF THE PARTICIPANT

I've read the information about the study. I've had enough opportunity to ask doubts/questions about it and I was answered to my satisfaction. I here by consent voluntarily to be a participant in this study, to respond to questionnaire, to undergo tests and measurements. I understand that I've the right to withdraw from the study or refuse to answer any of the questions.

PARTICIPANT'S NAME: .... DATE: ....

PARTICIPANT'S SIGNATURE/
THUMB IMPRESSION

FAMILY MEMBER'S NAME: ....

RELATION TO PARTICIPANT: ....

FAMILY MEMBER'S SIGNATURE/
THUMB IMPRESSION WITNESS NAME and SIGNATURE/
THUMB IMPRESSION

सहमात पत

शुरूआत

मेरा नाम .... है , सामुदाायक ाचाकत्सा ावभाग, आईएमएस, बीएचयू, वाराणसी से. हम chiraigaon ब्लाक म एक अध्ययन के संचालन कर रहे ह सीखने और बेहतर पोषण गभवता माहलाआ और वाराणसी का ग्रामाण आबादा म अपने प्रभाव का आकलन द्वारा और 100 आइएफए सेवन के ालए आहार प्रथाआ और 100 गभवता माहलाआ ाटप्स (द्वारा आइएफए सेवन सुधार). इस अध्ययन म भागादारा पूरा तरह स्वाच्छक है. हालााक हम बहुत ज्याद इस अध्ययन म आपका भागादारा का सराहना करते ह, करग के बाद से अपने ावचार बहुत महत्वपूण ह.

अध्ययन के ावाध

यह 3 घर यात्राआ और एक काद्रत समूह चचा (FGD) शाामल ह. 1 यात्रा (इस यात्रा) म, म गभावस्था के दौरान आप और आपके देखभाल, आहार और 100 आइएफए सेवन पारवार के सदस्या के बारे म पूछने जा रहा हूँ. प्रश्नावला के बारे म आमतौर पर 30-40 ामनट लगते ह को पूरा कर. अंत म हम अपनी बाइ अनाामका के ालए एक छाटा सी चुभन से खून का एक छाटा सी बूंद लेने के ालए एचबी (% लौह स्तर) के स्तर को मापने का तरह माप के रूप म अपनी गभवता माहलाआ म बहुत महत्वपूण होता है. प्राक्रया तुम बहुत कम परेशानी का कारण होना चााहए. लाकन यह पूरा तरह से आपके हमारे नमूना लेने के ालए अनुमात देने का फैसला है. पराक्षण के पारणाम 30-40 सेकंड के भीतर समझाया जाएगा. नमूना अध्ययन के बाद नष्ट हो जाएगा और ाकसा भी अन्य अनुसंधान म इस्तमाल नहा ाकया जाएगा. इसके अलावा, हम आपका वजन और ऊंचाई का माप लेने का तरह, अपने स्वास्थ्य और पोषण का ास्थात का आकलन करग. आप के ालए प्रश्नावल का इसी ावषय पर एक FGD म इतना भाग है ाक हम एक बेहतर तराका म अपनी धारणा और व्यवहार को समझ सकता हूँ का अनुरोध कर रहे ह.

2 यात्रा म हम आपको कुछ सरल तराका के बारे म ाशाक्षत करने के ालए अपने स्वास्थ्य ास्थात म सुधार होगा. आप केवल चुानदा और 12 सप्ताह का अवाध म एक मनमाने ढंग से बाहर करने का कााशश कुछ या सब के सब होगा. पराक्षण अवाध के अंत म हम 3 यात्रा के ालए आने के ालए पराक्षण के अपने अनुभव के बारे म पूछग. इसके अलावा हम (अपनी बाइ अनाामका के ालए एक छाटा सी चुभन द्वारा) एचबी% के दोहराने का माप ले, वजन और ऊंचाई का तरह होगा.

सभाावत लाभ

अध्ययन म आपका भागादारा म मदद ामलगा हम वतमान स्वास्थ्य और गभवता माहलाआ के पोषण

संबंधी ।स्थात और तुम उसी के बारे म ।शाक्षत करने के आकलन के ।लए. पराक्षण से अनुभव म मदद ।मलगा आप अपने स्वास्थ्य और पोषण का ।स्थात म सुधार के ।लए इतना है ।क तुम एक शारारक और मानासक रूप से स्वस्थ बच्च को पुन: पेश कर सकग. और आप अन्य गभवता माहलाआ के साथ अपने अनुभव को साझा कर पराक्षण ।कया जाएगा. पराक्षण के अनुभव से आपका प्रातााक्रया के ।लए समुदाय म गभवता माहलाआ का भलाई के ।लए स्वस्थ संदेश का प्रसार ।कया जाएगा.

क्षमत खतरे

कोई समाावत खतरा इस अध्ययन म शाामल ह. हम ।डस्पाजबल lancets (सुई) का उपयोग करने के ।कसा भी प्रकार के सक्रमण से बचने के द्वारा सभी सड़न रोकनेवाला सावधानी बरतनी होगी.

गोपनीयता

अध्ययन म आपका भागादारा के ।वषय म जानकारा रखा पूण और कानून ।सफ वज्ञाानक उद्दश्य के ।लए इस्तमाल का अनुमात दा हद तक गोपनीय जाएगा. अनुसंधान दल के सदस्या के अलावा कोई भी पराक्षा पारणाम के ।लए उपयोग होगा. आपका नाम ।कसा भी ।रपाट म इस्तमाल नहा ।कया जाएगा या ।कसा भी रूप म जारा ।कया जाएगा.

वापस लेने के अध्ययन से आधकार

आप ।कसा भी या सभी सवाला के जवाब देने से मना आधकार है, के अध्ययन के दौरान ।कसा भी पराक्षा या माप गुज़रना पड़ता है. तुम भी ।कसा भी समय इस अध्ययन से वापस लेने का आधकार है. लाकन हम आशा है ।क तुम पूरा तरह से अध्ययन म भाग लग, के रूप म आपके ।वचार महत्वपूण ह.

कृपया मुझे अगर म कुछ भी स्पष्ट कहा गया है और म समझाने का यह सुानाश्चत करने के ।लए आगे तुम समझ खुशी होगी नहा पता है.

बताया भाग लेने का अनुमात

म इस अध्ययन के बारे म जानकारा पढ़ा है. म करने के ।लए पयाप्त संदेह पूछने का मौका ।मला है / इसके बारे म और म अपनी सतुाष्ट के सवाला का जवाब था. सहमात यहाँ म ने स्वच्छ से इस अध्ययन म भागीदार बनने के ।लए, को प्रश्नाव का जवाब है, पराक्षण और माप गुज़रना पड़ता है. म समझता हूँ ।क म इस अध्ययन से वापस लेने या ।कसा भी सवाल का जवाब इंकार करने का आधकार है.

'भागीदार नाम: .... ।दनाक: ....

'भागीदार हस्ताक्ष / अंगूठे का छाप
