# Supplementary material for: Trials of Improved Practices (TIPs) to Enhance the Dietary and Iron-Folate Intake during Pregnancy- A Quasi Experimental Study among Rural Pregnant Women of Varanasi, India
Source: PLoS One. 2015 Sep 14;10(9):e0137735. doi: 10.1371/journal.pone.0137735 (PMC4569533; doi:10.1371/journal.pone.0137735)
Supplement: S2 File — (PDF) [file pone.0137735.s002.pdf]

FACULTY OF MEDICINE
INSTITUTE OF MEDICAL SCIENCES
BANARAS HINDU UNIVERSITY

No.Dean/2010-11/ 83. Dated: 5-5-2010

The Head,
Department of Community Medicine
Institute of Medical Sciences
Banaras Hindu University

Dear Sir,

The Ethical Committee meeting was held on 04.05.2010 at 2.00 p.m in the Chamber of the undersigned for ethical clearance of the thesis proposed for MD course in your department.

Thesis Title of Dr. Siddharudha Shivalli – Study of effectiveness of TIPs (Trails of improved practices) methodology against maternal anemia in chiraigaon block of Varanasi District
Ethical Observation- .
Remarks: The above synopsis has been approved by the ethical committee

This is for your information and necessary action at your end.

(B.D.BHATIA)
DEAN &
COORDINATOR

Yours sincerely,

(USHA KIRAN MEHRA)
CHAIRPERSON OF THE ETHICAL COMMITTEE
